# Cognitive Mechanisms Between Psychosocial Resources and the Behavioral Intention of Professional Help-Seeking for Internet Gaming Disorder Among Chinese Adolescent Gamers: Cross-Sectional Mediation Study

**DOI:** 10.2196/52478

**Published:** 2024-11-14

**Authors:** Yanqiu Yu, Joyce Hoi-Yuk Ng, Ji-bin Li, Jianxin Zhang, Joseph T F Lau

**Affiliations:** 1 School of Public Health, Fudan University, Shanghai, China; 2 Centre for Health Behaviours Research, Jockey Club School of Public Health and Primary Care, The Chinese University of Hong Kong, Hong Kong, China (Hong Kong); 3 Department of Clinical Research, State Key Laboratory of Oncology in South China, Collaborative Innovation Center for Cancer Medicine, Sun Yat-sen University Cancer Center, Guangzhou, China; 4 West China School of Public Health, Sichuan University, Chengdu, China; 5 Zhejiang Provincial Clinical Research Center for Mental Disorders, The Affiliated Wenzhou Kangning Hospital, Wenzhou Medical University, Wenzhou, China; 6 Public Mental Health Center, School of Mental Health, Wenzhou Medical University, Wenzhou, China

**Keywords:** professional help-seeking, behavioral intention, internet gaming disorder, IGD, perception, China, mental health, psychosocial resource, secondary school, gamer, cross-sectional survey

## Abstract

**Background:**

Internet gaming disorder (IGD) is a global public health concern for adolescents due to its potential severe negative consequences. Professional help-seeking is important for early screening, diagnosis, and treatment of IGD. However, research on the factors associated with professional help-seeking for IGD as well as relevant mediation mechanisms among adolescents is limited.

**Objective:**

Based on the stress coping theory, the conservation of resource theory, and behavioral change theories, this study investigated the prevalence and factors influencing the behavioral intention of professional help-seeking for internet gaming disorder (BI-PHSIGD). The research also explored the underlying mechanisms, including psychosocial resources like resilience and social support, perceived resource loss due to reduced gaming time, and self-efficacy, in professional help-seeking among adolescent internet gamers.

**Methods:**

A cross-sectional survey was conducted among secondary school students who were internet gamers in 2 Chinese cities from October 2019 to January 2020. Data from the full sample (N=1526) and a subsample of 256 IGD cases (according to the 9-item *DSM-5* [*Diagnostic and Statistical Manual of Mental Disorders, Fifth Edition*] IGD Checklist) were analyzed. Multivariate logistic regression analysis was conducted to examine the factors of BI-PHSIGD, while structural equation modeling was performed to test the proposed mediation mechanisms.

**Results:**

The prevalence of BI-PHSIGD was 54.3% (829/1526) in the full sample and 40.6% (104/256) in the IGD subsample (vs 708/1239, 57.1% among non-IGD cases). In the full sample, psychosocial resources of resilience (adjusted odds ratio [aOR] 1.03, 95% CI 1.02-1.05) and social support (aOR 1.03, 95% CI 1.02-1.04) as well as self-efficacy in professional help-seeking (aOR 1.64, 95% CI 1.49-1.81) were positively associated with BI-PHSIGD, while perceived resource loss due to reduced gaming time was negatively associated with BI-PHSIGD (aOR 0.97, 95% CI 0.96-0.98); the positive association between psychosocial resources and BI-PHSIGD was fully mediated via 2 single-mediator indirect paths (via self-efficacy in professional help-seeking alone: effect size=53.4%; indirect effect/total effect=0.10/0.19 and via perceived resource loss due to reduced gaming time alone: effect size=17.8%; indirect effect/total effect=0.03/0.19) and one 2-mediator serial indirect path (first via perceived resource loss due to reduced gaming time then via self-efficacy in professional help-seeking: effect size=4.7%; indirect effect/total effect=0.009/0.19). In the IGD subgroup, a full mediation via self-efficacy in professional help-seeking alone but not the other 2 indirect paths was statistically significant.

**Conclusions:**

Many adolescent internet gamers, especially those with IGD, were unwilling to seek professional help; as a result, early treatment is often difficult to achieve. To increase BI-PHSIGD, enhancing psychosocial resources such as resilience and social support, perceived resource loss due to reduced gaming time, and self-efficacy in professional help-seeking may be effective. Future longitudinal and intervention studies are needed to confirm and extend the findings.

## Introduction

Adolescent internet gaming disorder (IGD) is a global health concern. It was positively associated with various negative consequences such as depression, academic performance, poor family relationships, and worsened social relationships [1,2]. Gaming disorder, including IGD and other forms of problematic gaming behaviors (eg, offline gaming and console gaming), was added to the *International Classification of Diseases, 11th Revision* (*ICD-11*) by the World Health Organization in 2019 [3], which officially defines and classifies IGD as a medical condition (ie, the medicalization of IGD). Recent reviews have identified a number of efficacious intervention methods for reducing IGD symptoms, such as cognitive behavioral therapy, multidimensional family therapy, and brief gaming abstinence [4-6]. Although a previous study showed that the awareness of IGD medicalization raised adolescents’ willingness to seek help to deal with IGD [7], it may intensify stigma related to professional help-seeking [8-10]. Researchers have called for more studies investigating the prevalence and factors of professional help-seeking for early prevention, diagnosis, and treatment of IGD [11].

It is warranted to promote the behavioral intention of professional help-seeking for internet gaming disorder (BI-PHSIGD). Theoretically and empirically, behavioral intention strongly predicts the behavior of concern [12,13]. The construct has been applied to investigate addictive behaviors [13]. In the literature, only 3 studies have been conducted about professional help-seeking for IGD. A longitudinal study reported the prevalence of BI-PHSIGD at 36% among general Chinese college students; factors associated with BI-PHSIGD included probable IGD, perceived resources, and illness perceptions of IGD [14]. A Swedish study and a Korean study reported factors of professional help-seeking behaviors for IGD, including perceived stigma, IGD diagnosis, the number of years of education, gaming time, quantities of online or offline friends, using gaming as a means of achievement or coping, and self-recognition of gaming problems [15,16]. These studied factors in general focused on gaming characteristics and cognitive factors.

According to the stress coping theory, an individual’s coping resources would affect his or her coping responses [17]. In response to a stressor, the availability of coping resources would affect stress appraisal, coping behavior or responses, and health outcomes [17]. Professional help-seeking regarding IGD can be seen as a positive coping response to some IGD-related stressors such as symptoms, academic problems, and family conflicts [18]. Psychosocial resources are an important domain of coping resources; they refer to the strengths and support derived from psychological factors (such as resilience) and social factors (such as support systems like family and friends) that enhance one’s ability to cope with stress and improve health outcomes [19,20]. Psychosocial resources hence cover both intrapersonal (psychological) resources and interpersonal (social) resources [19]. Resilience is a typical type of intrapersonal resource; it refers to the psychological capacity to recover from difficult life events [21], and it is associated with professional help-seeking regarding mental disorders among adolescents and young adults [22]. Social support, an interpersonal resource, refers to assistance derived from an individual’s social network [23]. Perceived social support is as important as enacted social support in reducing risk behaviors [24], improving mental health [25], and fostering help-seeking behaviors [22]. To our knowledge, no studies looked at the associations between these 2 types of psychosocial resources and professional help-seeking regarding IGD.

It is important to understand the cognitive mediators between psychosocial resources and BI-PHSIGD, one of which is perceived resource loss due to reduced gaming time. Resource loss is a key construct of the conservation of resource (COR) theory, postulating that actual or perceived resource losses could generate stress, which would in turn lead to negative psychological or behavioral outcomes [20,26-28]. Understandably, gaming time was positively associated with perceived rewards of improved self-esteem and social acceptance [29,30]; hence, reducing gaming time would imply potential losses in such rewards. A recent study developed and validated the resource losses due to the reduction in gaming time scale in Chinese adolescent gamers to assess related perceptions [31]; it revealed 2 domains of perceived personal resource losses (eg, loss in a sense of achievement) and perceived interpersonal resource losses (eg, loss in the number of friends), both of which were associated with a lower level of the behavioral intention to reduce gaming time [31]. According to the COR theory and empirical evidence, it was hence assumed that perceived resource losses due to reduced gaming time would be negatively associated with the behavioral outcome of BI-PHSIGD. In addition, the “loss spirals” concept of the COR theory postulates that those initially having fewer resources are more likely to lose (more) resources in the future, triggering a cascading effect [20,26-28]. Accordingly, individuals possessing more psychosocial resources (resilience and social support) may be less likely to perceive resource losses due to reduced gaming time. To our knowledge, no studies have investigated this association specifically, but there was indirect support. An empirical study reported that social support enhanced the sense of self-value among people with internet addiction and lowered the reliance on the internet to derive rewards [32]. It is hence plausible that those with more psychosocial resources might rely less on using internet gaming to generate resources and then tend to have a lower level of perceived resource losses due to reduced gaming time. Given the above, it was hypothesized that psychosocial resources of resilience and social support would be associated with a lower level of perceived resource losses due to reduced gaming time, which in turn would be associated with a higher level of BI-PHSIGD (a mediation mechanism).

Cognitive factors are another potential mediator between psychosocial resources and BI-PHSIGD. Numerous behavioral change theories (eg, the social cognitive theory) and empirical studies highlight the cognitive factor of self-efficacy as one of the strongest predictors of health behaviors [33-36]. Self-efficacy refers to an individual’s belief in his or her capacity to perform a behavior [37]; it is an important factor of service use [38]. Accordingly, it was assumed that self-efficacy in professional help-seeking would be positively associated with BI-PHSIGD. In addition, the associations between psychosocial resources or perceived resource losses due to reduced gaming time and self-efficacy in professional help-seeking could be supported by the above “loss spirals” of the COR theory that those initially with more resources (eg, more psychosocial resources or lower levels of perceived resource losses) tend to gain more resources (eg, self-efficacy as an intrapersonal resource) in the future [20,26-28]. There was also empirical support; longitudinal studies reported that psychosocial resources of resilience and social support were positively associated with self-efficacy [39,40]. Notably, due to the novelty of the variable of perceived resource losses due to reduced gaming time, no relevant empirical studies were located, and this study could fill up this knowledge gap. Given the above, it was hypothesized that the positive associations between psychosocial resources would be mediated (1) via self-efficacy in professional help-seeking alone and (2) first via perceived resource losses due to reduced gaming time and then via self-efficacy in professional help-seeking.

This study investigated the level of BI-PHSIGD among secondary school students who played internet games in the past 12 months (internet gamers) in 2 Chinese cities (Chengdu and Guangzhou). It tested three potential factors of BI-PHSIGD: (1) psychosocial resources (ie, resilience and social support), (2) perceived resource losses due to reduced gaming time, and (3) self-efficacy in professional help-seeking. In addition, this study tested the mediation effects of perceived resource losses due to reduced gaming time and self-efficacy in professional help-seeking on the association between psychosocial resources and BI-PHSIGD. Specifically, the following hypotheses were tested:

- Hypothesis 1: Psychosocial resources of both resilience and social support would be positively associated with BI-PHSIGD.
- Hypothesis 2: Perceived resource losses due to reduced gaming time would be negatively associated with BI-PHSIGD.
- Hypothesis 3: Self-efficacy in professional help-seeking would be positively associated with BI-PHSIGD.
- Hypothesis 4: Psychosocial resources of resilience and social support would be associated with a lower level of perceived resource losses due to reduced gaming time, which in turn would be associated with a higher level of BI-PHSIGD (a single-mediator indirect path).
- Hypothesis 5: Psychosocial resources of resilience and social support would be associated with a higher level of self-efficacy in professional help-seeking, which in turn would be associated with a higher level of BI-PHSIGD (a single-mediator indirect path).
- Hypothesis 6: Psychosocial resources of resilience and social support would be associated with a lower level of perceived resource losses due to reduced gaming time, which would in turn be associated with a higher level of self-efficacy in professional help-seeking that would be associated with a higher level of BI-PHSIGD (a 2-mediator indirect path).

Furthermore, the above research hypotheses was further tested in adolescents with IGD, as BI-PHSIGD may be more relevant to those at higher risk of IGD than to those at lower risk.

## Methods

### Participants and Data Collection

A cross-sectional survey was conducted among junior middle school students from October 2019 to January 2020 in Guangzhou and Chengdu, China. The 2 cities had population sizes of 15.3 and 16.6 million in 2019, respectively. With the coordination of local education sectors, 4 schools from Guangzhou and 3 from Chengdu were selected using convenience sampling. Within these schools, cluster sampling was adopted by inviting all grade 8 (8 years of formal education) students from the Guangzhou schools and all grade 7‐9 students from the Chengdu schools to participate. The self-administered, anonymous survey was conducted in the classroom setting in paper and pencil in the absence of schoolteachers. The data collection procedure was the same as in previous studies [41,42]. Prior to data collection, trained fieldworkers briefed the students about the voluntary nature of this study, the logistics, and the right to quit at any stage. It was mentioned by the fieldworker and printed on the cover page of the questionnaire that the return of the completed questionnaire would imply the provision of informed consent. The fieldworkers assisted with inquiries from students and did the quality check during and after the students submitted the questionnaires, which took about 40 minutes to complete.

There were 3039 completed questionnaires, among which 60 (2%) were removed, as such questionnaires had missing values in more than 20% (9/46) of all the items. A total of 1272 (41.9%) were removed, as the participants had not played internet games in the past 12 months; 181 (6%) were further removed due to no response to the key variables (BI-PHSIGD). The remaining 1526 were used for data analysis (in Guangzhou: n=779, 51% and in Chengdu: n=747, 49%).

### Ethical Considerations

This study conducted an anonymous, self-administered survey among adolescents without identifiable information. The informed consent was obtained via 3 sources. First, school consent was obtained from school principals before data collection. Second, parents were informed about the survey, and they had the right to refuse their children’s participation. Third, the participating students were clearly informed by the fieldworker that this study was anonymous and voluntary to participate and that submission of the completed would indicate the provision of informed consent; the information was clearly printed on the cover page of the questionnaire. Notably, no written informed consent was collected to maintain anonymity. No incentive was provided to any party involved during data collection. This study, including the procedures of participant recruitment and informed consent collection, was approved by the Survey and Behavioural Research Ethics Committee of the Chinese University of Hong Kong in 2019 (SBRE-18‐430).

### Measurements

#### Behavioral Intention of Professional Help-Seeking for Internet Gaming Disorder

Two items assessed whether the participants would seek professional help from school counselors or clinical mental health professionals (eg, psychologists and psychiatrists) among those with IGD at present or in the future (yes or no response options). The binary dependent variable of BI-PHSIGD was defined as the willingness to seek help from either 1 of the 2 types of professionals.

#### Internet Gaming Disorder

It was assessed by using the 9-item *DSM-5* (*Diagnostic and Statistical Manual of Mental Disorders, Fifth Edition*) IGD Checklist [43]. An IGD case was defined when endorsing at least 5 of the 9 *DSM-5* symptoms (preoccupation, withdrawal, tolerance, inability to control internet gaming, prioritization, continuation despite adverse consequences, deception, avoidance, and significant loss) in the past 12 months (yes or no response options). The Chinese version has been validated among adolescents and showed satisfactory psychometric properties [44]. The Cronbach α of the scale was 0.79 in this study.

#### Resilience

It was assessed by using the 10-item Connor-Davidson Resilience Scale [21]. The Chinese version has been validated among adolescents and showed satisfactory psychometric properties [45]. A sample item is “I can adapt to change.” The items were rated on a 5-point Likert scale (0=never to 4=always). Higher scores indicated higher levels of resilience. The Cronbach α of the scale was 0.92 in this study.

#### Perceived Social Support

It was assessed by two 4-item subscales of the Multidimensional Scale of Perceived Social Support (8 items in total), rating the levels of perceived social support from family members and friends, respectively [23]. Its Chinese version has been validated among adolescents and showed acceptable psychometric properties [46]. A sample item is “My family members or friends really try to help me.” The items were rated on a 7-point Likert scale (1=extremely disagree to 7=extremely agree); higher scores indicated higher levels of perceived social support. The Cronbach α of the scale was 0.93 in this study.

#### Perceived Resource Losses Due to Reduced Gaming Time

It was assessed by the 11-item personal resource loss subscale of the resource loss due to reduction in gaming time scale, which has been developed and validated among Chinese adolescents with satisfactory psychometric properties [31]. A sample item is “Reducing gaming time makes me lose a sense of achievement.” The items were rated on a 5-point Likert scale (0=no loss at all to 4=loss to an extremely great extent); higher scores indicated higher levels of perceived resource losses due to reduced gaming time. The Cronbach α of the scale was 0.96 in this study.

#### Self-Efficacy in Professional Help-Seeking

The item “If you were now having IGD and intend to seek help from health professionals, to what extent are you capable of doing so?” (1=none at all to 5=extremely capable) was used.

#### Sociodemographics

Information was collected about age (years), sex, whether living with both parents, single-parent family, and perceived household financial situations relative to classmates.

### Statistical Analysis

Chi-square test was performed to examine the associations between the categorical variables. Pearson correlation coefficients were generated among the variables of interest. Two similar sets of analyses were conducted for the full sample and the IGD subsample, respectively. Multivariate logistic regression was conducted to test the associations between the psychosocial or cognitive variables and BI-PHSIGD after adjusting for sociodemographics.

Structural equation modeling (SEM) was performed to test the underlying mechanisms between psychosocial resources and BI-PHSIGD via mediators of perceived resource losses due to reduced gaming time and self-efficacy in professional help-seeking after adjusting for sociodemographics. The weighted least square mean and variance estimator was used in SEM, considering that BI-PHSIGD was treated as a binary dependent variable. A latent variable of psychosocial resources was derived from scale scores of resilience and social support. The goodness-of-fit of SEM was indicated by Comparative Fit Index≥0.90 and root mean square error of approximation ≤0.08 [47,48]. Standardized path coefficients (β) were reported. The mediation effect was assessed by using a bootstrapping method (n=2000); the indirect effect would be considered significant if the 95% CI did not involve 0. The effect size of the significant mediation effects was the proportion of the total effect explained by the specific indirect path. SPSS (version 23.0; IBM Corp) and Mplus (version 7.0; Muthén & Muthén) were used for statistical analyses; 2-tailed *P*<.05 were considered statistically significant.

## Results

### Descriptive Statistics

The results are shown in Table 1. In the full sample, the mean age was 13.5 (SD 0.8; range 10‐19) years. Over half were male (910/1526, 59.6%) participants; about one-fifth were not living with both parents (319/1526, 20.9%) and came from a single-parent family (252/1526, 16.5%). The prevalence of IGD was 16.7% (256/1526). The IGD group (n=256) was more likely than the non-IGD group (n=1239) to include participants who were older, male, living in Chengdu, and not living with both parents. The prevalence of BI-PHSIGD in the full sample was 54.3% (829/1526), which was lower in the IGD group than in the non-IGD group (104/256, 40.6% vs 708/1239, 57.1%; *P*<.001). Of those who intended to seek professional help in the full sample, 79.7% (661/829) would like to see psychologists or psychiatrists, and 55.9% (464/829) would like to see school counselors (the data were not tabulated). The mean (SD; range) scores of resilience, social support, perceived resource losses due to reduced gaming time, and self-efficacy in professional help-seeking in the full sample and the IGD subsample are presented in Table 2.

**Table 1.** Descriptive statistics of sociodemographics and BI-PHSIGD^a^ in the full sample and IGD^b^ subsample of Chinese adolescents in a cross-sectional survey in 2019.

	The full sample (n=1526)	IGD case		
		Yes (n=256)	No (n=1239)	*P* value
**Sociodemographics**				
Age (years), mean (SD) (range 10‐19)	13.5 (0.8)	13.6 (0.8)	13.5 (0.8)	.006
**Studied sites, n (%)**				<.001
Guangzhou	779 (51)	76 (29.7)	700 (56.5)	
Chengdu	747 (49)	180 (70.3)	539 (43.5)	
**Sex, n (%)**				<.001
Female	607 (39.8)	75 (29.3)	515 (41.6)	
Male	910 (59.6)	177 (69.1)	719 (58)	
Missing data	9 (0.6)	4 (1.6)	5 (0.4)	
**Living with both parents, n (%)**				.02
Yes	1197 (78.4)	186 (72.7)	243 (19.6)	
No	319 (20.9)	66 (25.8)	990 (79.9)	
Missing data	10 (0.7)	4 (1.5)	6 (0.5)	
**Single-parent family, n (%)**				.44
No	1263 (82.8)	207 (80.9)	196 (15.8)	
Yes	252 (16.5)	45 (17.6)	1038 (83.8)	
Missing data	11 (0.7)	4 (1.5)	5 (0.4)	
Better household financial situation, mean (SD) (range 1‐5)	3.1 (0.8)	3.0 (0.9)	3.2 (0.7)	.06
**BI-PHSIGD**				<.001
No	697 (45.7)	152 (59.4)	531 (42.9)	
Yes	829 (54.3)	104 (40.6)	708 (57.1)	

a BI-PHSIGD: behavioral intention of professional help-seeking for internet gaming disorder.

b IGD: internet gaming disorder.

**Table 2.** Descriptive statistics and correlation analyses among the key variables in the full sample and IGD^a^ subsample of Chinese adolescents in a cross-sectional survey in 2019.

	Mean (SD)	Range	Resilience		Social support		Perceived resource losses due to reduced gaming time	
			*r*	*P* value	*r*	*P* value	*r*	*P* value
**The full sample (n=1526)**								
Resilience	23.2 (8.7)	0‐40	—^b^	—	—	—	—	—
Social support	38.5 (11.8)	8‐56	0.52	<.001	—	—	—	—
Perceived resource losses due to reduced gaming time	8.8 (9.6)	0‐44	−0.20	<.001	−0.28	<.001	—	—
Self-efficacy in professional help-seeking	3.6 (1.2)	1-5	0.34	<.001	0.32	<.001	−0.25	<.001
**The IGD subsample (n=256)**								
Resilience	22.1 (8.1)	0‐40	—	—	—	—	—	—
Social support	37.3 (11.1)	8-56	0.36	<.001	—	—	—	—
Perceived resource losses due to reduced gaming time	18.1 (10.0)	0‐44	0.18	.005	−0.11	.09	—	—
Self-efficacy in professional help-seeking	3.5 (1.1)	1-5	0.21	.001	0.15	.02	<0.001	.93

a IGD: internet gaming disorder.

b Not applicable.

### Correlation Analysis Among the Independent and Mediating Variables

The results are shown in Table 2. In the full sample, resilience, social support, and self-efficacy in professional help-seeking were positively correlated with each other (*r*=0.32-0.52). All these variables were negatively correlated with PRL-RGT (*r*=−0.28 to −0.20). Similar significant correlations were found in the IGD subgroup, except that the associations between social support or self-efficacy in professional help-seeking and perceived resource losses due to reduced gaming time were statistically nonsignificant.

### Factors of BI-PHSIGD

The results of the adjusted multivariate logistic regression analysis are shown in Table 3. In the full sample, resilience (adjusted odds ratio [aOR] 1.03, 95% CI 1.02-1.05), social support (aOR 1.03, 95% CI 1.02-1.04), and self-efficacy in professional help-seeking (aOR 1.64, 95% CI 1.49-1.81) were all positively associated with BI-PHSIGD, while perceived resource losses due to reduced gaming time was negatively associated with BI-PHSIGD (aOR 0.97, 95% CI 0.96-0.98). In the IGD subsample, similar significant associations were found between perceived resource losses due to reduced gaming time or self-efficacy in professional help-seeking and BI-PHSIGD, but the 2 associations between resilience or social support and BI-PHSIGD were statistically nonsignificant (Table 3).

**Table 3.** Factors of BI-PHSIGD^a^ in the full sample and IGD^b^ subsample of Chinese adolescents in a cross-sectional survey in 2019^c^.

	BI-PHSIGD (dependent variable)	
	The full sample (n=1526), aOR^d^ (95% CI)	IGD cases (n=256), aOR (95% CI)
Resilience	1.03 (1.02-1.05)	1.01 (0.98-1.05)
Social support	1.03 (1.02-1.04)	1.02 (0.99-1.04)
Perceived resource losses due to reduced gaming time	0.97 (0.96-0.98)	0.95 (0.92-0.98)
Self-efficacy in professional help-seeking	1.64 (1.49-1.81)	1.90 (1.47-2.46)

a BI-PHSIGD: behavioral intention of professional help-seeking for internet gaming disorder.

b IGD: internet gaming disorder.

c The models were adjusted for sociodemographics, including age, studied site, sex, whether living with both parents, single-parent family, and relative household financial situation.

d aOR: adjusted odds ratio.

### SEM Results

#### The Full Sample

**Figure 1.**
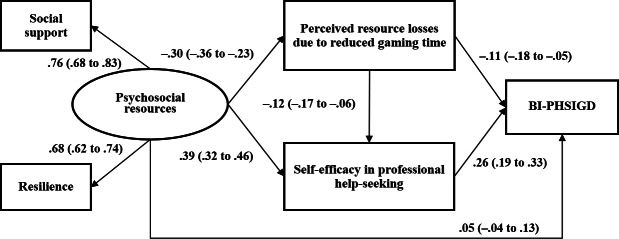
Structural equation modeling in the full sample of Chinese adolescents in a cross-sectional survey in 2019. Standard coefficients were reported; the model was adjusted for sociodemographics. BI-PHSIGD: behavioral intention of professional help-seeking for internet gaming disorder.

In Figure 1, the SEM model showed acceptable goodness-of-fit (Comparative Fit Index 0.91; root mean square error of approximation 0.07). The results support all the initial hypotheses, showing that the latent variables of psychosocial resources had significant indirect effects on BI-PHSIGD via three indirect paths: (1) a single-mediator path of better psychosocial resources → lower perceived resource losses due to reduced gaming time → better BI-PHSIGD (β=.03; 95% CI 0.01-0.05; *P*=.001; effect size=17.8%; indirect effect/total effect=0.03/0.19), that is, psychosocial resources were negatively associated with perceived resource losses due to reduced gaming time, which in turn was negatively associated with BI-PHSIGD; (2) a single-mediator path of better psychosocial resources → higher self-efficacy in professional help-seeking → lower BI-PHSIGD (β=.10; 95% CI 0.07-0.13; *P*<.001; effect size=53.4%; indirect effect/total effect=0.10/0.19), that is, psychosocial resources were positively associated with self-efficacy in professional help-seeking, which in turn was negatively associated with BI-PHSIGD; and (3) a serial 2-mediator path of psychosocial resources → perceived resource losses due to reduced gaming time → self-efficacy in professional help-seeking → BI-PHSIGD (β=.009; 95% CI 0.004-0.013; *P*<.001; effect size=4.7%; indirect effect/total effect=0.009/0.19), that is, psychosocial resources were negatively associated with perceived resource losses due to reduced gaming time that was negatively associated with self-efficacy in professional help-seeking, which in turn was positively associated with BI-PHSIGD. The direct effect of psychosocial resources on BI-PHSIGD was statistically nonsignificant, indicating that the above 3 indirect paths fully mediated the association between psychosocial resources and BI-PHSIGD.

#### The IGD Subsample

**Figure 2.**
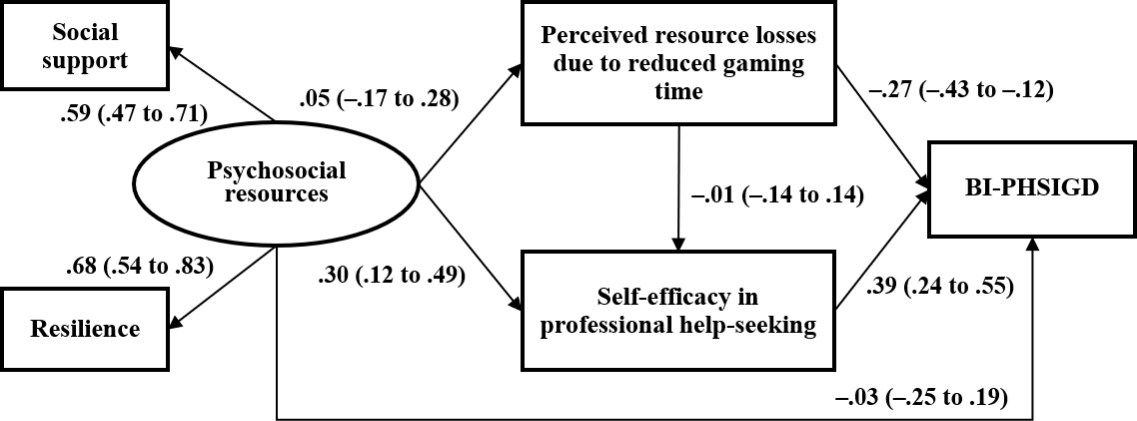
Structural equation modeling in the internet gaming disorder subsample of Chinese adolescents in a cross-sectional survey in 2019. Standard coefficients were reported; the model was adjusted for sociodemographics. BI-PHSIGD: behavioral intention of professional help-seeking in the case of internet gaming disorder.

In the IGD subsample, Figure 2 shows that the single-mediator path of better psychosocial resources → self-efficacy in professional help-seeking → BI-PHSIGD (β=.12; 95% CI 0.02-0.22; *P*=.02) was statistically significant and represented a full mediation. The other 2 indirect paths of better psychosocial resource → higher perceived resource losses due to reduced gaming time → lower BI-PHSIGD (β=−.02; 95% CI −0.08 to 0.05; *P*=.65) and psychosocial resource → perceived resource losses due to reduced gaming time → self-efficacy in professional help-seeking → BI-PHSIGD (β=.01; 95% CI −0.01 to 0.01; *P*=.99) were statistically nonsignificant.

## Discussion

### Principal Findings and Comparison to Prior Work

The observed prevalence of IGD among adolescent internet gamers was 16.7% (256/1526), corroborating similar Chinese studies (1084/6379, 17%) [49]. It was higher than that reported in studies conducted in Germany (35/1001, 3.5%) [50] and Thailand (1084/6379, 9.2%) [51], which used the same assessment tool and cutoff value as this study. Country variations have been observed in extant studies. For instance, Asian studies tended to report higher IGD prevalence [52]. Consistent with previous studies, male sex [53] and not living with both parents [54] were risk factors of IGD. Given the high prevalence and severe harms of IGD [1], interventions are warranted and should target the high-risk sociodemographic groups. Notably, China has implemented strict regulations to limit the amount of time on internet gaming among minors, aiming at preventing and controlling IGD among Chinese adolescents [55]. Whether such a policy would have beneficial effects on adolescents’ gaming behaviors, physical and mental health, and even broader societal implications has not gone through evaluation and greatly warrants investigation.

The prevalence of BI-PHSIGD in the full sample was 54.3% (829/1526); direct comparisons across regions cannot be made due to a lack of relevant studies among adolescents. Notably, in this study, such prevalence was lower in the IGD group than that in the non-IGD group (104/256, 40.6% vs 708/1239, 57.1%); such a difference was interesting and could be attributed to several reasons. First, similar to other mental disorders such as depression, there might be stigma related to seeking help for IGD after the medicalization of IGD [7]. IGD cases might feel ashamed of their ability to control their gaming behaviors, resulting in the reluctance to professional help-seeking due to fear of judgment or embarrassment. Second, IGD cases may not acknowledge the severity of their gaming problems or related impacts on their lives. Such a denial might lead to a lower level of BI-PHSIGD. It is a limitation of this study that it did not include questions about the IGD cases’ awareness and agreement regarding their IGD condition. Finally, IGD cases may see professional help-seeking as having to reduce gaming time substantially, which means more losses in gaming rewards. This speculation was supported by the higher level of perceived resource losses due to reduced gaming time in the IGD subgroup (mean 18.1, SD 10.0 vs mean 8.8, SD 9.6 among non-IGD cases) in this study. These may lead to a lower level of BI-PHSIGD. Nonetheless, the results suggest that the majority of those with IGD might not obtain professional help; the situation could be far from ideal and warrant intervention. In China, with the growing awareness of the importance of mental health and the unique needs of adolescents, the government recognizes the need to improve mental health services and integrates mental health into public health policies. For instance, an increasing number of hospitals and specialized mental health institutions have offered mental health services for adolescents, while some schools have started to incorporate mental health education and services into their curricula. However, despite progress, China’s adolescent mental health services face several challenges including a shortage of trained mental health professionals, high cost of professional services, and regional disparities in service availability. These could also be true for the case of professional help-seeking for IGD and should be taken into account in future intervention programs.

This study also found that psychosocial resources of resilience and social support were positively associated with BI-PHSIGD in the full sample of adolescent internet gamers. The finding supports our hypotheses derived from the stress coping theory [17] and corroborates previous empirical studies [22]. In the SEM, this positive association was found to be mediated via some mediators in both the full sample and the IGD subsample. Similarly, in both samples, both perceived resource losses due to reduced gaming time and self-efficacy in professional help-seeking were significantly associated with BI-PHSIGD. These results support the COR theory (resource losses would affect the behavioral outcomes) [20,26-28], numerous behavioral change theories (self-efficacy was a strong determinant of the behavior of concern) [33], and relevant empirical findings [31,34-36]. Notably, although the above findings were unable to test the mentioned theories directly, they shed some insights on the theoretical application to understanding professional help-seeking regarding IGD.

In the full sample, the mediation hypotheses were supported, and full mediations via perceived resource losses due to reduced gaming time and self-efficacy in professional help-seeking were found; previous studies also found significant associations among psychosocial resources, perceived resource losses, self-efficacy, and behavioral intention [35,36,39,40,56]. The indirect paths involving self-efficacy in professional help-seeking alone showed the largest effect size, followed by the mediation involving perceived resource losses alone, and then the serial 2-mediator indirect path having a relatively small effect size. It further suggests that self-efficacy is one of the most influential and modifiable determinants of behavioral changes [34]. According to the findings, to enlarge the potential beneficial effect of psychosocial resources on BI-PHSIGD, relevant interventions may promote self-efficacy in professional help-seeking and reduce perceived resource losses due to reduced gaming time, with the former possibly more efficacious than the latter.

Interventions attempting to increase BI-PHSIGD should pay special attention to the IGD cases who have a stronger need to seek professional help. The SEM of the IGD subsample gave results that were slightly different from those of the full sample: only the mediation via self-efficacy in professional help alone (a full mediation) was significant, but the other 2 direct paths involving perceived resource losses due to reduced gaming time were statistically nonsignificant. Again, the potentially important role of self-efficacy on the association between psychosocial resources and BI-PHSIGD is restated. The nonsignificant indirect effects in the IGD subgroup may be associated with the nonsignificant correlations between perceived resource losses due to reduced gaming time and social support or self-efficacy in professional help-seeking. The social relationships of IGD cases might have been strained or damaged due to their gaming behaviors [43]. This could mean that the impact of social support on their decision-making is reduced. In contrast, general gamers might still have robust social networks playing a more important role in influencing their perceptions of resource losses. On the other hand, the nonsignificant correlation between perceived resource losses due to reduced gaming time and self-efficacy in professional help-seeking suggests that such self-efficacy might not be strongly tied to these resource losses in IGD cases. For individuals with IGD, gaming is deeply embedded in their daily life and identity; hence, their confidence in seeking professional help might be more dependent on whether and how much they could handle the severe negative consequences (eg, health and social functions) rather than resource losses due to reduced gaming time. In contrast, gaming might be important but not central to the identity of general gamers, and in this case, perceived resource losses due to reduced gaming time are more likely to affect their self-efficacy in professional gaming following the “loss spirals” of the COR theory, as mentioned in the Introduction section. Nonetheless, these speculations need to be verified in future studies.

Overall, effective interventions promoting BI-PHSIGD may consider modifying the levels of psychosocial resources, perceived resource losses due to reduced gaming time, and self-efficacy in professional help-seeking. A recent review summarized some effective intervention components that can mobilize social support among adolescents, including explaining the benefits of social support, modeling friendly relationships, and improving social interaction skills [57]. Another review summarized effective interventions enhancing resilience in youths, including cognitive behavioral therapy with or without additional components (eg, art therapy), parenting skills intervention, and brief psychoeducation intervention [58]. Modification of perceived resource losses due to reduced gaming time can also be achieved via cognitive restructuring [59]. The promotion of self-efficacy can be achieved through relevant skill training (eg, emotional arousal and vicarious learning) [60].

This study was subjected to several limitations. First, as this was a cross-sectional study, temporal or causal inferences cannot be made. Future longitudinal and intervention studies are warranted to verify the findings. Second, reporting bias (eg, recall bias and social desirability bias) may exist, as the questionnaire was self-administered. For instance, students might be reluctant to disclose their IGD symptoms, and it is socially desirable to indicate BI-PHSIGD. Notably, the prevalence of IGD may be overestimated, as the *DSM-5* IGD Checklist tends to be a screening, instead of a diagnostic, tool for research purposes, and the response options failed to capture the frequencies of the IGD symptoms. Third, as the study population was conveniently selected from 7 junior middle schools in 2 Chinese cities, generalization of the results to other age groups, populations, and regions needs to be cautious. Furthermore, multivariate logistic regression analysis and SEM did not account for potential nested designs and intraclass correlation coefficients at the school level, although the study city was controlled. Fourth, as self-efficacy tends to be behavior-specific and there is a lack of relevant scales, a single item was constructed to assess self-efficacy in professional help-seeking; future studies are recommended to develop and validate multiitem or multidimensional scales of self-efficacy in this regard to confirm the current findings. Likewise, BI-PHSIGD was assessed by a single question, although similar single items have been used in numerous publications investigating behavioral intention [13,14]. Fifth, the readers should be reminded that the behavioral intention does not equate to the actual behavior, as a meta-analysis found that around a quarter of those with the behavioral intention would perform the actual behavior [61]. In addition, this study focused on professional help-seeking, while the other informal sources of help-seeking were not included (eg, friends and parents). Sixth, the effect size of the association between psychosocial resources of resilience and social support and BI-PHSIGD was small in this study; various psychosocial resources (eg, intrapersonal resources of self-esteem and interpersonal resources of social networks) might have stronger effects on BI-PHSIGD and should be investigated by future studies. Finally, structural factors such as the availability and affordability of the services might moderate the observed associations and should be considered in future relevant studies.

### Conclusions

This study observed the relatively low prevalence of BI-PHSIGD among adolescent internet gamers, which was even lower among the IGD cases than the non-IGD cases. Several factors of BI-PHSIGD were identified, including psychosocial resources of social support and resilience and cognitions of perceived resource losses due to reduced gaming time and self-efficacy in professional help-seeking. Furthermore, the positive association between psychosocial resources and BI-PHSIGD was fully mediated via self-efficacy in professional help-seeking in the IGD subgroup, and this indirect path showed the largest effect size in the full sample. Future longitudinal and intervention studies are required to confirm these findings.

## Abbreviations

aOR: adjusted odds ratio
BI-PHSIGD: behavioral intention of professional help-seeking for internet gaming disorder
COR: conservation of resource
DSM-5: Diagnostic and Statistical Manual of Mental Disorders, Fifth Edition
*ICD-11*: *International Classification of Diseases, 11th Revision*
IGD: internet gaming disorder
SEM: structural equation modeling
